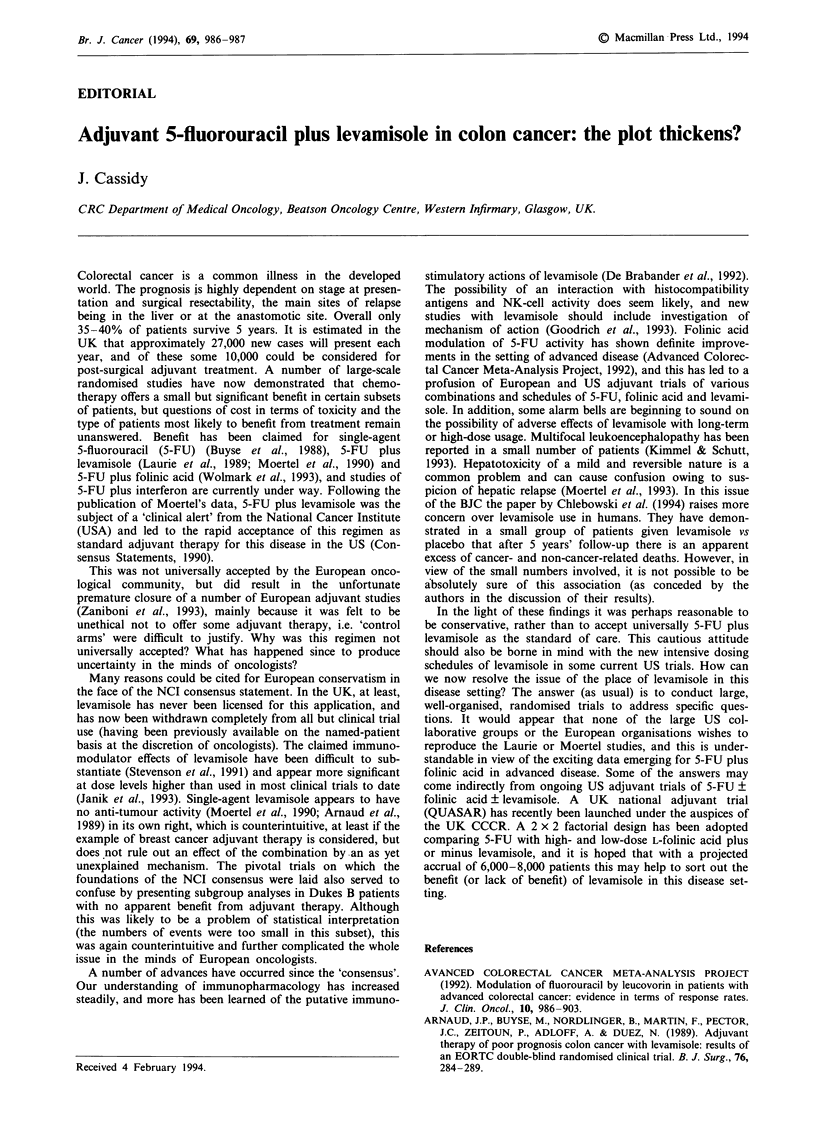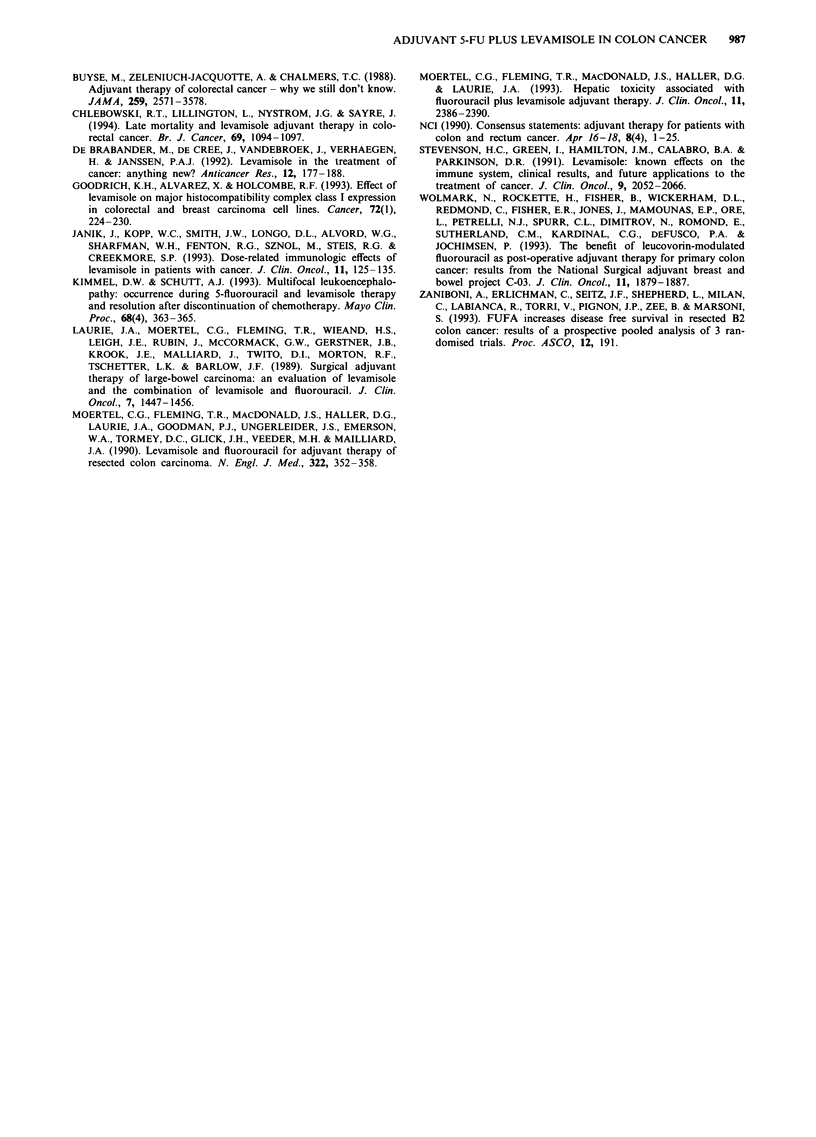# Adjuvant 5-fluorouracil plus levamisole in colon cancer: the plot thickens?

**DOI:** 10.1038/bjc.1994.193

**Published:** 1994-06

**Authors:** J. Cassidy


 © Macmillan Press Ltd., 1994

EDITORIAL

### Adjuvant 5-fluorouracil plus levamisole in colon cancer: the plot thickens?

J. Cassidy

*CRC Department of Medical Oncology, Beatson Oncology Centre, Western Infirmary, Glasgow, UK.*

Colorectal cancer is a common illness in the developed world. The prognosis is highly dependent on stage at presentation and surgical resectability, the main sites of relapse being in the liver or at the anastomotic site. Overall only 35–40% of patients survive 5 years. It is estimated in the UK that approximately 27,000 new cases will present each year, and of these some 10,000 could be considered for post-surgical adjuvant treatment. A number of large-scale randomised studies have now demonstrated that chemotherapy offers a small but significant benefit in certain subsets of patients, but questions of cost in terms of toxicity and the type of patients most likely to benefit from treatment remain unanswered. Benefit has been claimed for single-agent 5-fluorouracil (5-FU) (Buyse *et al.*, 1988), 5-FU plus levamisole (Laurie *et al.*, 1989; Moertel *et al.*, 1990) and 5-FU plus folinic acid (Wolmark *et al.*, 1993), and studies of 5-FU plus interferon are currently under way. Following the publication of Moertel's data, 5-FU plus levamisole was the subject of a 'clinical alert' from the National Cancer Institute (USA) and led to the rapid acceptance of this regimen as standard adjuvant therapy for this disease in the US (Consensus Statements, 1990).

This was not universally accepted by the European oncological community, but did result in the unfortunate premature closure of a number of European adjuvant studies (Zaniboni *et al.*, 1993), mainly because it was felt to be unethical not to offer some adjuvant therapy, i.e. 'control arms' were difficult to justify. Why was this regimen not universally accepted? What has happened since to produce uncertainty in the minds of oncologists?

Many reasons could be cited for European conservatism in the face of the NCI consensus statement. In the UK, at least, levamisole has never been licensed for this application, and has now been withdrawn completely from all but clinical trial use (having been previously available on the named-patient basis at the discretion of oncologists). The claimed immunomodulator effects of levamisole have been difficult to substantiate (Stevenson *et al.*, 1991) and appear more significant at dose levels higher than used in most clinical trials to date (Janik *et al.*, 1993). Single-agent levamisole appears to have no anti-tumour activity (Moertel *et al.*, 1990; Arnaud *et al.*, 1989) in its own right, which is counterintuitive, at least if the example of breast cancer adjuvant therapy is considered, but does not rule out an effect of the combination by an as yet unexplained mechanism. The pivotal trials on which the foundations of the NCI consensus were laid also served to confuse by presenting subgroup analyses in Dukes B patients with no apparent benefit from adjuvant therapy. Although this was likely to be a problem of statistical interpretation (the numbers of events were too small in this subset), this was again counterintuitive and further complicated the whole issue in the minds of European oncologists.

A number of advances have occurred since the 'consensus'. Our understanding of immunopharmacology has increased steadily, and more has been learned of the putative immunostimulatory actions of levamisole (De Brabander *et al.*, 1992). The possibility of an interaction with histocompatibility antigens and NK-cell activity does seem likely, and new studies with levamisole should include investigation of mechanism of action (Goodrich *et al.*, 1993). Folinic acid modulation of 5-FU activity has shown definite improvements in the setting of advanced disease (Advanced Colorectal Cancer Meta-Analysis Project, 1992), and this has led to a profusion of European and US adjuvant trials of various combinations and schedules of 5-FU, folinic acid and levamisole. In addition, some alarm bells are beginning to sound on the possibility of adverse effects of levamisole with long-term or high-dose usage. Multifocal leukoencephalopathy has been reported in a small number of patients (Kimmel & Schutt, 1993). Hepatotoxicity of a mild and reversible nature is a common problem and can cause confusion owing to suspicion of hepatic relapse (Moertel *et al.*, 1993). In this issue of the BJC the paper by Chlebowski *et al.* (1994) raises more concern over levamisole use in humans. They have demonstrated in a small group of patients given levamisole *vs* placebo that after 5 years' follow-up there is an apparent excess of cancer- and non-cancer-related deaths. However, in view of the small numbers involved, it is not possible to be absolutely sure of this association (as conceded by the authors in the discussion of their results).

In the light of these findings it was perhaps reasonable to be conservative, rather than to accept universally 5-FU plus levamisole as the standard of care. This cautious attitude should also be borne in mind with the new intensive dosing schedules of levamisole in some current US trials. How can we now resolve the issue of the place of levamisole in this disease setting? The answer (as usual) is to conduct large, well-organised, randomised trials to address specific questions. It would appear that none of the large US collaborative groups or the European organisations wishes to reproduce the Laurie or Moertel studies, and this is understandable in view of the exciting data emerging for 5-FU plus folinic acid in advanced disease. Some of the answers may come indirectly from ongoing US adjuvant trials of 5-FU ± folinic acid ± levamisole. A UK national adjuvant trial (QUASAR) has recently been launched under the auspices of the UK CCCR. A 2 × 2 factorial design has been adopted comparing 5-FU with high- and low-dose L-folinic acid plus or minus levamisole, and it is hoped that with a projected accrual of 6,000–8,000 patients this may help to sort out the benefit (or lack of benefit) of levamisole in this disease setting.

Received 4 February 1994.

### References

AVANCED COLORECTAL CANCER META-ANALYSIS PROJECT (1992). Modulation of fluorouracil by leucovorin in patients with advanced colorectal cancer: evidence in terms of response rates. *J. Clin. Oncol.*, **10**, 986–903.

ARNAUD, J.P., BUYSE, M., NORDLINGER, B., MARTIN, F., PECTOR, J.C., ZEITOUN, P., ADLOFF, A. & DUEZ, N. (1989). Adjuvant therapy of poor prognosis colon cancer with levamisole: results of an EORTC double-blind randomised clinical trial. *B. J. Surg.*, **76**, 284–289.

BUYSE, M., ZELENIUCH-JACQUOTTE, A. & CHALMERS, T.C. (1988). Adjuvant therapy of colorectal cancer – why we still don't know. *JAMA*, **259**, 2571–3578.

CHLEBOWSKI, R.T., LILLINGTON, L., NYSTROM, J.G. & SAYRE, J. (1994). Late mortality and levamisole adjuvant therapy in colorectal cancer. *Br. J. Cancer*, **69**, 1094–1097.

DE BRABANDER, M., DE CREE, J., VANDEBROEK, J., VERHAEGEN, H. & JANSSEN, P.A.J. (1992). Levamisole in the treatment of cancer: anything new? *Anticancer Res.*, **12**, 177–188.

GOODRICH, K.H., ALVAREZ, X. & HOLCOMBE, R.F. (1993). Effect of levamisole on major histocompatibility complex class I expression in colorectal and breast carcinoma cell lines. *Cancer*, **72**(1), 224–230.

JANIK, J., KOPP, W.C., SMITH, J.W., LONGO, D.L., ALVORD, W.G., SHARFMAN, W.H., FENTON, R.G., SZNOL, M., STEIS, R.G. & CREEKMORE, S.P. (1993). Dose-related immunologic effects of levamisole in patients with cancer. *J. Clin. Oncol.*, **11**, 125–135.

KIMMEL, D.W. & SCHUTT, A.J. (1993). Multifocal leukoencephalopathy: occurrence during 5-fluorouracil and levamisole therapy and resolution after discontinuation of chemotherapy. *Mayo Clin. Proc.*, **68**(4), 363–365.

LAURIE, J.A., MOERTEL, C.G., FLEMING, T.R., WIEAND, H.S., LEIGH, J.E., RUBIN, J., MCCORMACK, G.W., GERSTNER, J.B., KROOK, J.E., MALLIARD, J., TWITO, D.I., MORTON, R.F., TSCHETTER, L.K. & BARLOW, J.F. (1989). Surgical adjuvant therapy of large-bowel carcinoma: an evaluation of levamisole and the combination of levamisole and fluorouracil. *J. Clin. Oncol.*, **7**, 1447–1456.

MOERTEL, C.G., FLEMING, T.R., MACDONALD, J.S., HALLER, D.G., LAURIE, J.A., GOODMAN, P.J., UNGERLEIDER, J.S., EMERSON, W.A., TORMEY, D.C., GLICK, J.H., VEEDER, M.H. & MAILLIARD, J.A. (1990). Levamisole and fluorouracil for adjuvant therapy of resected colon carcinoma. *N. Engl. J. Med.*, **322**, 352–358.

MOERTEL, C.G., FLEMING, T.R., MACDONALD, J.S., HALLER, D.G. & LAURIE, J.A. (1993). Hepatic toxicity associated with fluorouracil plus levamisole adjuvant therapy. *J. Clin. Oncol.*, **11**, 2386–2390.

NCI (1990). Consensus statements: adjuvant therapy for patients with colon and rectum cancer. *Apr 16–18*, **8**(4), 1–25.

STEVENSON, H.C., GREEN, I., HAMILTON, J.M., CALABRO, B.A. & PARKINSON, D.R. (1991). Levamisole: known effects on the immune system, clinical results, and future applications to the treatment of cancer. *J. Clin. Oncol.*, **9**, 2052–2066.

WOLMARK, N., ROCKETTE, H., FISHER, B., WICKERHAM, D.L., REDMOND, C., FISHER, E.R., JONES, J., MAMOUNAS, E.P., ORE, L., PETRELLI, N.J., SPURR, C.L., DIMITROV, N., ROMOND, E., SUTHERLAND, C.M., KARDINAL, C.G., DEFUSCO, P.A. & JOCHIMSEN, P. (1993). The benefit of leucovorin-modulated fluorouracil as post-operative adjuvant therapy for primary colon cancer: results from the National Surgical adjuvant breast and bowel project C-03. *J. Clin. Oncol.*, **11**, 1879–1887.

ZANIBONI, A., ERLICHMAN, C., SEITZ, J.F., SHEPHERD, L., MILAN, C., LABIANCA, R., TORRI, V., PIGNON, J.P., ZEE, B. & MARSONI, S. (1993). FUFA increases disease free survival in resected B2 colon cancer: results of a prospective pooled analysis of 3 randomised trials. *Proc. ASCO*, **12**, 191.